# Functional buffering via cell-specific gene expression promotes tissue homeostasis and cancer robustness

**DOI:** 10.1038/s41598-022-06813-4

**Published:** 2022-02-22

**Authors:** Hao-Kuen Lin, Jen-Hao Cheng, Chia-Chou Wu, Feng-Shu Hsieh, Carolyn Dunlap, Sheng-hong Chen

**Affiliations:** 1 Lab for Cell Dynamics, Institute of Molecular Biology, Academia Sinica, Taipei, 115 Taiwan; 2 College of Medicine, National Taiwan University, Taipei, 106 Taiwan; 3 Genome and Systems Biology Degree Program, Academia Sinica and National Taiwan University, Taipei, Taiwan

**Keywords:** Genetic interaction, Systems analysis, Genome informatics, Cancer genomics, Cell biology, Computational biology and bioinformatics, Systems biology

## Abstract

Functional buffering that ensures biological robustness is critical for maintaining tissue homeostasis, organismal survival, and evolution of novelty. However, the mechanism underlying functional buffering, particularly in multicellular organisms, remains largely elusive. Here, we proposed that functional buffering can be mediated via expression of buffering genes in specific cells and tissues, by which we named Cell-specific Expression-BUffering (CEBU). We developed an inference index (C-score) for CEBU by computing C-scores across 684 human cell lines using genome-wide CRISPR screens and transcriptomic RNA-seq. We report that C-score-identified putative buffering gene pairs are enriched for members of the same duplicated gene family, pathway, and protein complex. Furthermore, CEBU is especially prevalent in tissues of low regenerative capacity (e.g., bone and neuronal tissues) and is weakest in highly regenerative blood cells, linking functional buffering to tissue regeneration. Clinically, the buffering capacity enabled by CEBU can help predict patient survival for multiple cancers. Our results suggest CEBU as a potential buffering mechanism contributing to tissue homeostasis and cancer robustness in humans.

## Introduction

Robustness in biological systems is critical for organisms to carry out vital functions in the face of environmental challenges^1,2^. A fundamental requirement for achieving biological robustness is functional buffering, whereby the biological functions performed by one gene can also be attained via other buffering genes. Although functional buffering has long been regarded as a critical function contributing to biological robustness, the mechanisms underlying functional buffering remain largely unclear^3^. Based on transcriptional regulation of buffering genes, functional buffering can be categorized as either needs-based buffering or intrinsic buffering. Needs-based buffering involves transcriptional activation of buffering genes only when the function of a buffered gene is compromised. To accomplish needs-based buffering, a control system must exist to detect compromised function and then activates expression of buffering genes. Needs-based buffering is often observed as genetic compensation in various biological systems including fungi, animals and plants^3–6^. One classical needs-based buffering mechanism is genetic compensation among duplicated genes, whereby expression of a paralogous gene is upregulated when the function of the active duplicated gene is compromised^4^. Genetic analyses of duplicated genes in *Saccharomyces cerevisiae* have revealed upregulation of gene expression in ~ 10% of paralogs when cell growth is compromised due to deletions of their duplicated genes^6–8^. Apart from duplicated genes, non-orthologous/analogous genes can also be activated for needs-based buffering^9^. For instance, inactivation of one growth signaling pathway can lead to activation of others for the coordination of cell growth and survival^3,4^. Such needs-based buffering genes have been documented as enabling unicellular/multicellular organisms to cope with environmental stresses^3,8^.

Recent genome-wide studies of duplicated genes in human cells have revealed another class of buffering mechanism whereby expression of buffering genes is not responsive to impaired function but is constitutively expressed, hereafter termed “intrinsic buffering”^10–12^. In some duplicated gene families, the strength of paralogous gene expression determines the essentiality of their corresponding duplicated genes in human cell lines, i.e., the higher the expression of paralogous genes in a particular cell line, the less essential are their duplicated genes^10–12^. This observation indicates that paralogs may buffer and contribute to the function of their duplicated genes in specific cells through their constitutive gene expression. In addition to duplicated gene families, gene essentiality can depend on inherent variability in the expression levels of other genes in the same pathway, suggesting that functionally analogous genes in the same pathway can also buffer each other^13^. Despite these observations, it remains unclear what mechanism may give rise to this context-dependent constitutive expression of buffering genes and how such intrinsic buffering may function in multicellular organisms.

**Figure 1:**
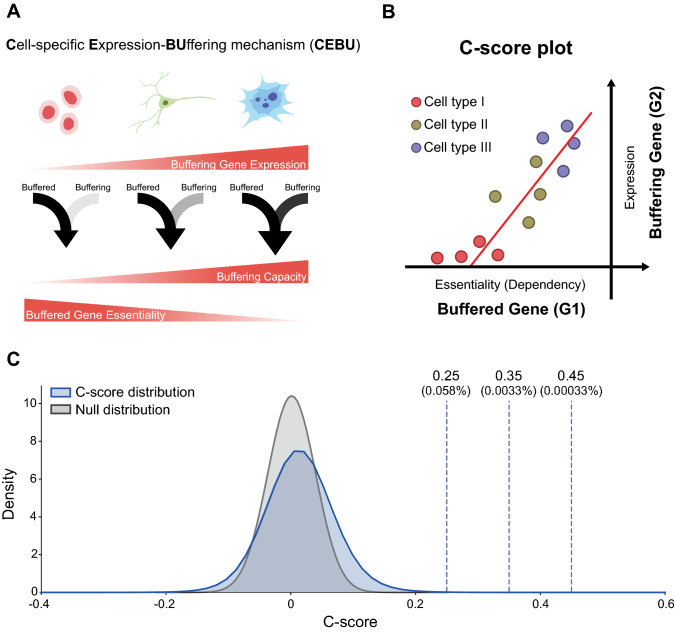
Genome-wide CEBU analysis using the C-score index. (**A**) Illustration of the cell-specific expression-buffering mechanism (CEBU). Cells with higher buffering gene (G2) expression (darker arrow) have stronger buffering potential, thereby lowering the essentiality of the buffered gene (G1). The illustration and cartoons are created using Adobe Illustrator. (**B**) C-score plot: the x-axis is the dependency score of the buffered gene (G1) and the y-axis is the expression level of the buffering gene (G2). G1 is considered as being potentially buffered by G2, as quantified by C-score, which is an adjusted correlation for a given gene based on the C-score plot. (**C**) Distribution of C-scores for all gene pairs (blue) and one of five randomly shuffled distributions (grey). The plot shows C-score between 0.4 and 0.6. The dashed lines indicate the C-score percentiles of 0.25, 0.35 and 0.45 for the all-gene-pair distribution.

In this study, we investigated if cell- and tissue-specific gene expression can act as an intrinsic buffering mechanism to buffer functionally related genes in the genome, thereby strengthening cellular plasticity for cell- and tissue-specific tasks. We proposed this concept as the “Cell-specific Expression-BUffering” or CEBU mechanism (illustrated in Fig. 1A). To explore CEBU as a potential buffering mechanism and to estimate buffering capability, we developed an inference index, the C-score, to identify putative gene pairs displaying CEBU. This index calculates the adjusted correlation between expression of a buffering gene and the essentiality of the buffered gene (Fig. 1B), utilizing transcriptomics data^14^ and genome-wide dependency data from the DepMap project^14,15^ across 684 human cell lines. Our results suggest the potential authenticity of the CEBU mechanism and this CEBU-mediated intrinsic buffering may play a critical role in cell-specific survival, tissue homeostasis, and cancer robustness.

## Results

### Development of the C-score to infer cell-specific expression buffering (CEBU)

In seeking an index to explore intrinsic buffering operated via constitutive gene expression, we postulated a buffering relationship whereby the essentiality of a buffered gene (G1) increases when expression of its buffering gene (G2) decreases across different human cell lines (Fig. 1). Given that G2 expression differs among cell lines, the strength of buffering capacity varies across cell lines, thereby conferring on G1 cell-specific essentiality. This concept prompted the formulation of the cell-specific expression buffering mechanism, here named CEBU, which can be quantitatively investigated by developing an inference index, C-score. The C-score of a gene pair is derived from the correlation between the essentiality of a buffered gene (G1) and expression of its buffering gene (G2) (see C-score plot, Fig. 1B), and is formulated as:

$$\mathrm{C}{\text{-}}\mathrm{score }={\rho }_{G1,G2}\left(1+b\frac{{slope}_{min}}{{slope}_{G1,G2}}\right)$$

where *ρ* denotes the Pearson correlation coefficient between essentiality of G1 and expression of G2. Their regression slope (*slope*_*G1, G2*_) is normalized by *slope*_*min*_, which denotes the minimum slope of all considered gene pairs in the human genome (see “Methods”). The normalized slope can be weighted by cell- or tissue type-specific *b*. In our current analysis, *b* is set as 1 for a pan-cell- and pan-cancer-type analysis. Gene essentiality is represented by dependency scores (D.S.) from the DepMap project^15^, where the effect of each gene on cell proliferation was quantified after its knockout using the CRISPR/Cas-9 approach. Specifically, a more negative D.S. reflects slower cell proliferation when the gene is knocked out, thus reflecting stronger essentiality. Expression data was obtained through RNA-seq ^14^. We anticipated that the higher the C-score of a gene pair, the more likely G2 would buffer G1 based on our proposed CEBU mechanism.

We conducted a genome-wide analysis to calculate C-scores for all genes with negative mean dependency scores (G1s) pairing with expressed genes (G2s), yielding 9196 G1 × 13,577 G2 gene pairs across 684 human cell lines (Fig. 1C and see “Methods”). For our analysis, we considered gene pairs to have a high C-score with a strong likelihood of intrinsic buffering when their C-scores were > 0.25, yielding 64,439 gene pairs which comprises 0.058% of the 9196 × 13,577 gene pairs in the human genome (Supplementary Table S1 and Data availability). To assess whether the results are due to random chance, we generated a bootstrapped null distribution by random shuffling of G2 expression among cell lines (Fig. 1C). This null distribution can be modeled as a normal distribution (Supplementary Fig. S1), for which we determined the high C-score gene pairs are statistically significant with a *q*-value < 2.2e-16 after multiple testing correction (Fig. 1C). Table 1 lists the top 20 G1s pairing with the G2 that yields the highest C-score. The highest C-score gene pair is *FAM50A* and *FAM50B*, which are potential transcriptional regulators recently discovered to be synthetic lethal^16^. Other well described synthetic lethal cases include *RPP25L/RPP25*^16–18^, *CDS2/CDS1*^16^ (Table 1) as well as other high C-score gene pairs, *UAP1/UAP1L1*^16,19,20^, *SMARC4/SMARC2*^16,18,20,21^, and *ENO1/ENO2*^22^ (Supplementary Table S1). Comparing with nine other predictions of synthetic lethality^23–31^ to four larger scale studies of experimental evidences^16,19,20,32^, the high C-score gene pairs demonstrated among the highest number of matches to experiments, even though matches between prediction to experiments remains few (0 to 14.3%, Supplementary Table S2). This result indicate identification of buffering and synthetic lethality is non-trivial, possibly due to a wide variety of biological mechanisms, and the CEBU concept may be one of these mechanisms. Furthermore, based on the concept of CEBU, a high C-score should be indicative of marked variability in cell-specific essentiality and expression. Indeed, we observed higher variability in both G1 dependency and G2 expression for high C-score gene pairs (Supplementary Fig. S2A). Nevertheless, high variation alone is insufficient to grant a high C-score. A high C-score requires consistent pairing between G1 and G2 across cell lines and, as anticipated, disrupting the pairing between G1 dependency and G2 expression by shuffling G2 expression amongst cell lines (without changing variability) abolished the C-score relationship (compare the right and left panels of Supplementary Fig. S2B). Moreover, both mean G1 dependency and mean G2 expression in high C-score gene pairs were lower than those parameters in randomly selected gene pairs (Supplementary Fig. S2C), implying that G1s and G2s in high C-score gene pairs tend to be more essential and less expressed, respectively.

**Table 1:** Top 20 C-score gene pairs.

	G1	G2	C-score	Duplicated or non-duplicated
1	*FAM50A*	*FAM50B*	0.793	D
2	*RPP25L*	*RPP25*	0.770	D
3	*CDS2*	*CDS1*	0.677	D
4	*EFR3A*	*EFR3B*	0.657	D
5	*RAB6A*	*RAB6B*	0.639	D
6	*INTS6*	*INTS6L*	0.624	D
7	*CHMP4B*	*CHMP4C*	0.618	D
8	*ATP6V0E1*	*ATP6V0E2*	0.610	D
9	*TTC7A*	*TTC7B*	0.609	D
10	*DNAJC19*	*DNAJC15*	0.606	D
11	*STX4*	*STX2*	0.600	D
12	*IRF4*	*PTK2*	0.588	ND
13	*PCYT1A*	*PCYT1B*	0.578	D
14	*NMT1*	*NMT2*	0.562	D
15	*SNAP23*	*SNAP25*	0.552	D
16	*ATP1B3*	*ATP1B1*	0.547	D
17	*MYB*	*CAMSAP2*	0.547	ND
18	*MYBL2*	*MYBL1*	0.544	D
19	*TP53BP1*	*EDA2R*	0.540	ND
20	*POP7*	*RPP25*	0.537	ND

The top 20 G1s pairing with the G2 that yields the highest C-score is shown.

*D* duplicated gene pair, *ND* Non-duplicated gene pair.

The proposed CEBU mechanism describes an intrinsic buffering mechanism that functions under normal physiological conditions. Consistent with this notion, when we examined if the C-score index could be biased due to the usage of cancer cell lines, we found that only a low percentage (2.3% per gene pair, Supplementary Fig. S3A) of mutant cell lines contributed to our C-score measurements. Moreover, excluding mutant cell lines did not qualitatively affect our C-score measurements, especially for high C-score gene pairs (Supplementary Fig. S3B). The same trend holds for cancer-related genes (Supplementary Fig. S3B). These results indicate that mutant cell lines are not the major determinants of C-scores. Similarly, since copy number variation (CNV) is a major mechanism for oncogenic expression, we checked if CNV contributes to G2 expression. As shown in Supplementary Fig. S3C, the correlation between G2 expression and copy number decreases with increasing C-score, indicating that CNV is not a primary mechanism regulating G2 expression. Thus, the C-score index is likely not biased by the utilization of cancer cell lines. Moreover, genes located chromosomal adjacently in the same topological associating domain (TAD) tend to co-express^33^. Hence, we interrogated if G2 is adjacent to a duplicated gene of G1. We found only 15,582/64,439 (24.2%) G2s are on the same chromosome as any G1 duplicated gene. Within these, the minimum distances between a G2 to any G1 duplicated gene are generally greater than the typical size of a TAD (750 kbp^34^ to 2.5 Mbps^35^), indicating that G2 is not selected by adjacency to G1 duplicated gene (Supplementary Fig. S3D).

### Characterization of C-score-inferred CEBU gene pairs

**Figure 2:**
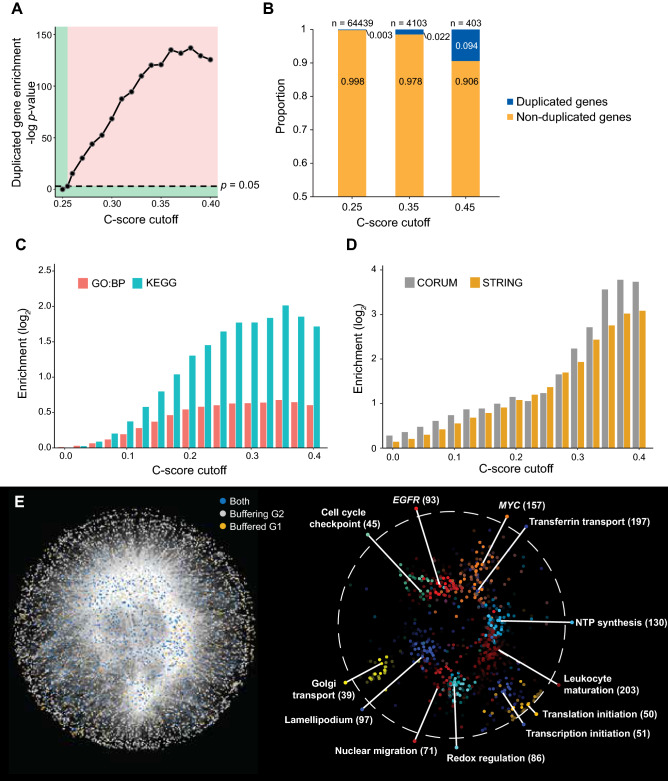
Properties of high C-score gene pairs. (**A**) Enrichment for duplicated genes as C-scores increase (hypergeometric test). The dashed line denotes *p*-value of 0.05, where the corresponding C-score is 0.255. The red region (i.e., above the dashed line and equating to C-score > 0.255) indicates significant enrichment. The green region indicates lack of significance. (**B**,**C**) Functional enrichment of C-score gene pairs increases with C-score cutoff. (**B**) Percentage of C-score-identified non-duplicated and duplicated gene pairs with different C-score cut-offs. (**C**) Enrichment of pairs of genes annotated with the same gene ontology biological process (GO:BP) term or KEGG pathway in C-score gene pairs. Enrichment increases with C-score cut-off. (**D**) Enrichment of pairs of genes with annotated protein–protein interactions from STRING and within the same protein complex from CORUM among C-score gene pairs. Enrichment increases with C-score cut-off. (**E**) Left: the buffering gene network is composed of 6664 nodes and 42,754 edges with C-scores > 0.2536 visualized using Cytoscape (https://cytoscape.org/). Orange nodes represent buffered genes; grey nodes are buffering genes, and blue nodes are genes that are both buffered and buffering. Right: Clusters of GO-enriched biological functions in the buffering gene network. Numbers indicate the number of genes in the cluster.

Since several duplicated gene pairs have been implicated as displaying functional buffering via gene expression^10–12^, we checked if C-score identified duplicated gene pairs are over-represented. Among the high C-score gene pairs, 210 pairs are duplicated gene pairs. We found that duplicated gene pairs are more enriched among high C-score gene pairs as C-score increases (Fig. 2A and see “Methods”), where statistically significant enrichment is reached at C-scores > 0.255 (*p*-value = 0.05 by using a hypergeometric test). This enrichment increases with higher C-scores suggests a higher likelihood that CEBU describes a potential buffering mechanism utilize by a portion of duplicated genes (Fig. 2A). Interestingly, the majority of high C-score gene pairs are non-duplicated (> 90%, Fig. 2B). In these cases, we asked if G2s may be functional analogs of the respective G1s, acting as surrogate genes. Accordingly, we examined if the high C-score gene pairs are more likely to participate in the same function or biological pathway or physically interact. We calculated the enrichment of curated gene sets in terms of Gene Ontology (GO)^36^ and Kyoto Encyclopedia of Genes and Genomes (KEGG)^37^ from the Molecular Signatures Database^38^ (Fig. 2C). Gene pairs with high C-scores consistently exhibited greater functional enrichment. Likewise, we observed a monotonic increase in the enrichment of protein–protein interactions (PPI) [using the STRING^39^ and CORUM^40^ databases] between G1s and G2s in accordance with increasing C-score cutoff (Fig. 2D). To gain insight into which functions are represented in the proposed CEBU mechanism, we considered that G1 may be paired with multiple G2s and vice versa, and constructed a C-score network consisting of G1s, G2s, and genes that act as both G1s and G2s (Fig. 2E). The enriched functions include housekeeping functions such as regulating redox homeostasis, gene transcription, mRNA translation, as well as NTP synthesis (Fig. 2E). Moreover, some cancer-related pathways are also enriched in the C-score-identified buffering network, including the proto-oncogenes *EGFR* and *MYC* (Fig. 2E). Both duplicated and non-duplicated gene pairs contribute to the observed functional and PPI enrichments. However, notably, the increase in functional and PPI enrichment as C-score increases are primarily attributable to non-duplicated genes as there is an observable increase in enrichment for all genes while enrichment for duplicated genes is relatively constant with increasing C-scores (compare Fig. 2C,D to Supplementary Fig. S4A,B). These results indicate a strong likelihood for intrinsic buffering among analogous genes in the same pathway or proteins in the same protein complex. Thus, high C-score gene pairs are enriched in duplicated gene pairs, as well as non-duplicated gene pairs that are members of the same biological pathway and/or encode physically interacting proteins, supporting that the proposed CEBU mechanism (which is the basis of the C-score index) may provide an explanation to a part of intrinsic buffering between such gene pairs.

### Experimental validation of C-score-inferred CEBU gene pairs

**Figure 3:**
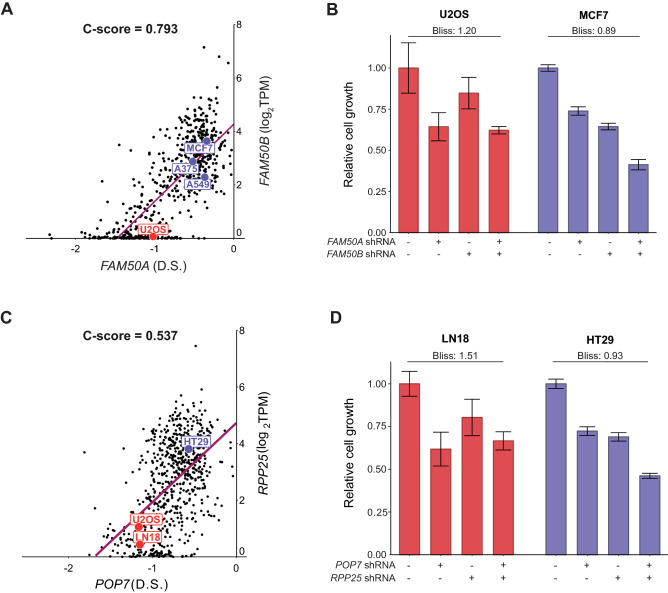
Experimental validation of cell-specific expression buffering between *FAM50A* and *FAM50B*, and *POP7* and *RPP25*. (**A**) C-score plot of the highest C-score gene pair, *FAM50A* (dependency score—D.S.) and *FAM50B* (G2) expression, labeled with the U2OS (predicted not synergistic) and MCF7 (predicted synergistic) cell lines. (**B**) Relative cell growth between 0 and 90 h based on fold-change in confluency of the U2OS and MCF7 cell lines with or without *FAM50A* or *FAM50B* suppression. Bliss scores indicate strength of synergy between double suppression of *FAM50A* and *FAM50B* compared to either gene alone. Error bars indicate standard deviation of six technical repeats. (**C**) C-score plot of the non-duplicated gene pair, i.e., *POP7* dependency score and *RPP25* gene expression, labeled with the LN18 (predicted not synergistic) and HT29 (predicted synergistic) cell lines. (**D**) Relative cell growth between 0 and 90 h based on fold-change in confluency of the HT29, U2OS and LN18 cell lines with or without shRNA-based *POP7* or *RPP25* suppression. Bliss scores indicate strength of synergy between double suppression of *POP7* and *RPP25* compared to either gene alone. Error bars indicate standard deviation of six technical repeats.

To validate putative C-score-inferred buffering gene pairs, we conducted experiments on the highest C-score gene pair, i.e., *FAM50A*–*FAM50B*, both members of which belong to the same duplicated gene family. Based on a C-score plot of *FAM50A–FAM50B* (Fig. 3A), we expected that *FAM50B* would display a stronger buffering effect on *FAM50A* for cell lines located at the top-right of the plot (e.g. MCF7) relative to those at the bottom-left (e.g. U2OS). Accordingly, growth of the cell lines at the top-right of the plot would be more sensitive to dual suppression of *FAM50A* and *FAM50B*. We used gene-specific small hairpin RNAs (shRNAs) to suppress expression of *FAM50A* and *FAM50B*, individually and in combination. The suppressive effects of the shRNAs were verified using RT-qPCR (Supplementary Fig. S5C). Consistent with our expectations, we observed stronger growth suppression in the MCF7 cell line relative to the U2OS cell line in two experimental repeats (Fig. 3B, Supplementary Fig. S5A,B). An additional cell line, A549, located at the middle of the C-score plot, however, showed a medium growth suppression relative to MCF7 and U2OS cells (Supplementary Fig. S5A,B). Next, we quantified *FAM50A* and *FAM50B* genetic interactions in these different cell lines by Bliss score^41^, where scores lower than one implies stronger synergistic interactions (see “Methods”). Indeed, the *FAM50A*–*FAM50B* gene pair in MCF7 cell lines was calculated to have a potential synergistic effect, whereas the calculation showed no synergistic effect in the U2OS cell line (Bliss score: 0.89 in MCF7 and 1.20 in U2OS). Importantly, a recent study focusing on genetic interaction of duplicated genes identified the *FAM50A* and *FAM50B* gene pair as the most significant interacting duplicated gene pair in the human genome^16^, further supporting the inference power of our C-score index. Moreover, the A375 cell line was used in that recent study, and it is predicted to display strong synergy based on our C-score plot of *FAM50A* and *FAM50B* (Fig. 3A)*.*

Although duplicated genes are well recognized for their buffering relationship, there is limited evidence supporting intrinsic buffering among non-duplicated genes. Thus, we sought to experimentally examine a pair of non-duplicated genes with a high C-score, so we targeted the *POP7*–*RPP25* pair. These two genes encode protein subunits of the ribonuclease P/MRP complex. In the C-score plot of *POP7*–*RPP25* (Fig. 3C), the HT29 cell line lies in the top-right region and the U2OS and LN18 cell lines are in the bottom-left region, inferring a likelihood for a stronger buffering effect in the HT29 cell line. To validate buffering effects, we suppressed expression of *POP7* and *RPP25* using gene-specific shRNAs in these three cell lines (verified using RT-qPCR, Supplementary Fig. S5F). Since expression of *RPP25* is too low to be quantified using RT-qPCR, we validated suppression of *RPP25* using western blot (Supplementary Fig. S6). Consistent with the C-score plots, we observed that dual suppression of *POP7* and *RPP25* corresponded with calculations of strong synergistic effects for the HT29 cell line but not for the U2OS or LN18 cell lines (Bliss scores for *POP7*–*RPP25* genetic interactions are 0.931 in HT29 and 1.51 in LN18, shown in Fig. 3D; and Bliss score of 1.03 in U2OS is shown in S5D. Experimental repeat shown in Supplementary Fig. S5E). Collectively, the results suggest that C-score-inferred buffering gene pairs can be non-duplicated functional analogs in the same protein complex or duplicated genes of the same family.

### Tissue specificity of CEBU

**Figure 4:**
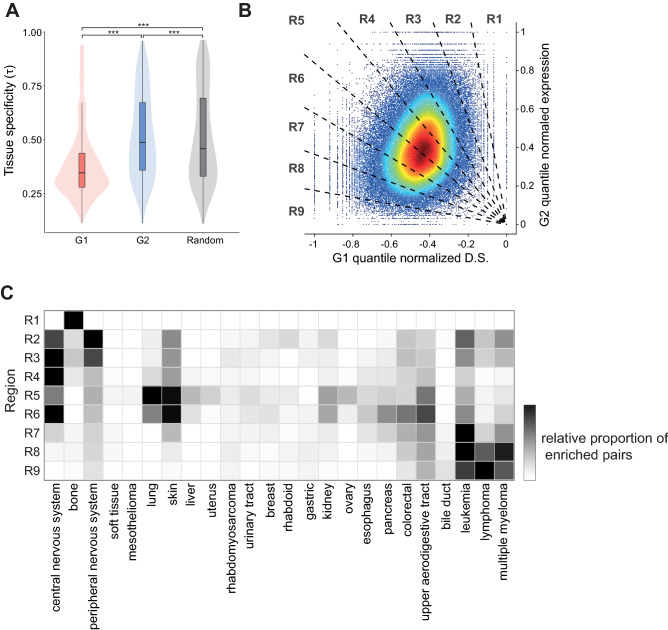
Tissue specificity of CEBU. (A) Tissue specificity (*τ*) of G1 and G2 pairs. *τ* was calculated for G1 and G2 from high C-score gene pairs. Statistical significance was assessed by paired-*t* test. (**B**) Density plot showing overlay of 100,000 randomly selected high C-score gene pairs. D.S. (G1s) and expression (G2s) were normalized to be between − 1 and 0 or 0 and 1, respectively. Each dot represents a cell line based on its normalized D.S. for G1 and normalized expression for G2. Color gradient indicates low to high density as blue to dark red, respectively. The normalized C-score plot was divided equally into nine regions (R1–R9) by radiating lines out from zero. (**C**) Tissue/cell-type specificity of each region (R1–R9) of the normalized C-score plot. The heatmap represent the relative percentage of statistically enriched gene pairs for the corresponding tissue/cell types. The heatmap is created by the Conditional Formatting function of Excel.

One key feature of intrinsic buffering is cross-cell variation in the expression of buffering genes (G2s) in conjunction with cell-specific dependency of the buffered genes (G1s) (Fig. 1). We hypothesized that the source of this cross-cell variation in G2 expression is embedded in the distinct transcriptional programs of different tissues. Therefore, we examined if the expression of high C-score G1s and G2s is tissue-specific. We calculated a tissue specificity index, *τ*^42^, for each gene to establish if it displays low (low *τ*, broadly expressed across tissues) or high tissue specificity (high *τ*, only expressed in one or a few specific tissues). As shown in Fig. 4A, G2s generally presented higher tissue specificity compared to G1s (significant with *t*-test, *p* < 2.2e−16) and compared to the control generated by randomly shuffling G2s across cell lines (Supplementary Fig. S7A). Together, these results indicate that G1s are generally expressed in the majority of cell types, whereas expression of G2s is more tissue-specific.

The pronounced tissue-specificity of G2 expression supports the cell-specific buffering concept proposed by CEBU and may be extended to tissue-specific intrinsic buffering. To further explore tissue distribution of the CEBU concept, we generated normalized C-score plots for all high C-score gene pairs whereby the G1 dependency scores across all cell lines were quantile-normalized to be between − 1 and 0 and the G2 expression values were normalized to be between 0 and 1 (Fig. 4B). We plotted these values against each other and then divided the resulting plot into nine equal regions by radiating lines out from zero (R1 to R9, Fig. 4B). As per the examples suggest in Fig. 3A,C, we hypothesized that there would be specific tissue types enriched in the regions R1–R4, which we speculated to display stronger CEBU-mediated buffering capacity. Similarly, there would be enrichment of specific tissue types in regions R6–R9, which we speculated to infer lower buffering capacity. Accordingly, considering a total of 29 tissue/cell types, we calculated the proportion of each tissue/cell type in each region of the plot in Fig. 4B, as well as the percentage of CEBU-enriched gene pairs for each tissue/cell type (see “Methods”). For each plot region, we indeed observed specific enrichment in one to three tissue/cell types presented a high percentage of CEBU-enriched gene pairs (Fig. 4C and Supplementary Fig. S7B). For example, for region R1, 98.0% of the CEBU-enriched gene pairs are highly expressed in cells derived from bone tissue (Fig. 4C), whereas region R9 encompasses a high percentage of strongly-expressing CEBU-enriched gene pairs in blood cells (lymphoma: 13.9%, leukemia: 10.6%, and multiple myeloma: 9.2% Fig. 4C). We also noted a few reoccurring tissue/cell types across regions speculated to infer high buffering capacity (central nervous system in R2, R3, and R4) or in low buffering regions (leukemia in R7, R8, and R9; lymphoma in R8 and R9) (Fig. 4C), indicating that particular tissue/cell types are with specific distributions given a CEBU setting. These results strengthen that CEBU may be capable of reflecting tissue-specific intrinsic buffering, and that whereas buffered G1s are generally expressed across tissue types, the buffering G2s are expressed in specific tissue/cell types, potentially contributing to tissue-specific functions.

### Harnessing C-score to calculate the buffering capacity of CEBU

**Figure 5:**
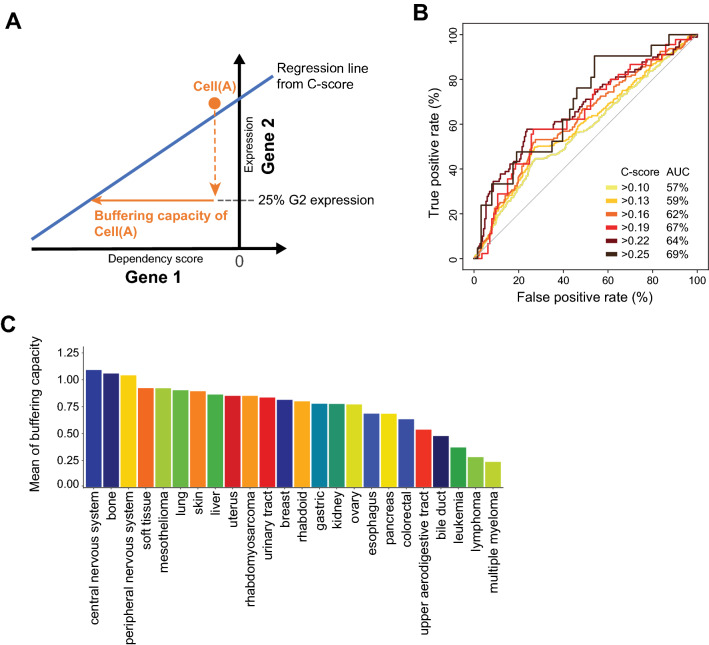
C-score-derived tissue-specific buffering capacity. (**A**) Illustration showing how cell-specific buffering capacities were derived from C-scores. Buffering capacity was calculated based on: (1) the regression line of the C-score for the gene pair; and (2) relative G2 expression (compared to that of all other cell lines) for the cell line of interest. See “Methods” for the formula for buffering capacity calculation. (**B**) Predictive performance shown as ROC curves for predicting genetic interactions using cell-specific buffering capacity. Prediction sets consist of 84 data-points (37 unique genetically interacting gene pairs) across 8 cell lines with a C-score cut-off of 0.25. (**C**) Mean buffering capacity for each tissue type (lower panel) and the corresponding proportion of enriched gene pairs for each region (upper panel).

As suggested by our experimental results in Fig. 3, cell lines located in the upper right of a C-score plot are more sensitive to dual gene suppression, indicating a possibility of higher buffering capacity from G2s. Adding to this notion, specific tissues tend to be enriched at the upper right of the C-score plot (Fig. 4), prompting investigation on cell/tissue-specific buffering capacity. To quantify G2 buffering capacities in various cells or tissues, we calculated buffering capacities as the relative G2 expression (compared to that of all other cell lines) of the cell line of interest adjusted by the C-score of the gene pair (Fig. 5A and “Methods”). We hypothesized that the C-score-derived buffering capacities can be an indirect inference of functional buffering and genetic interaction. Hence, we sought to validate these C-score-derived buffering capacities between G1 and G2 using experimental results from four independent studies in human cells (Supplementary Table S3)^19,32,43,44^. The four studies are described as follows: Rosenbluh et al. investigated the genetic interaction map of beta-catenin-active and beta-catenin-inactive cancers with combinatorial CRISPR screening in four cancer cell lines^43^. Shen et al. examined 73 gene pairs in combination (141,912 interactions) in three different cell lines^32^. With a more robust CRISPR screening approach, Najm et al. performed pairwise combinations of 158 genes knockout in six cancer cell lines^44^. Focusing on metabolic genes, Zhao et al. did a combinatorial CRISPR screening in two cell lines^19^. Using the receiver operating characteristic (ROC) curve to assess the performance of buffering capacity predictions, we observed that the resulting area under curve (AUC) is significantly larger than random (Mann–Whitney U test with false discovery rate, FDR < 0.1, Fig. 5B). Furthermore, predictive performance increased for higher C-score cutoffs, as indicated by their increasing AUC (Fig. 5B). Moreover, the buffering capacity is quantitatively correlated with the strength of genetic interaction. We observed a negative correlation between C-score-derived buffering capacities and experimentally validated genetic interactions (C-score cutoff = 0.25, correlation = − 0.231, *p*-value = 0.034, FDR = 0.081, Supplementary Fig. S8A), and this correlation is stronger for higher C-score cutoffs (Supplementary Fig. S8B). Even though this correlation coefficient of − 0.231 is not strong (although it is statistically significant), the intrinsic variability associated with collating experimental results from four independent studies must be considered a contributory factor to weakening that correlation^19,32,43,44^. Moreover, predictions of genetic interactions based on CEBU buffering capacity are robust even when different thresholds for calculating buffering capacity are applied (“Methods” and Supplementary Fig. S8C). Accordingly, CEBU buffering capacity may be used to infer genetic interactions in human cells. Since CEBU may be reflective of tissue-specific intrinsic buffering (Fig. 4), we also quantified buffering capacity in various tissue/cell types. We calculated the average buffering capacity for each tissue/cell type based on high C-score gene pairs (Fig. 5C). In line with our enrichment analysis presented in Fig. 4, the top three most buffered tissues are the central nervous system, bone and the peripheral nervous system. In contrast, blood cells—including multiple myeloma, lymphoma, and leukemia cell lines—exhibited the lowest buffering capacities.

### CEBU-mediated buffering capacity is indicative of cancer aggressiveness

**Figure 6:**
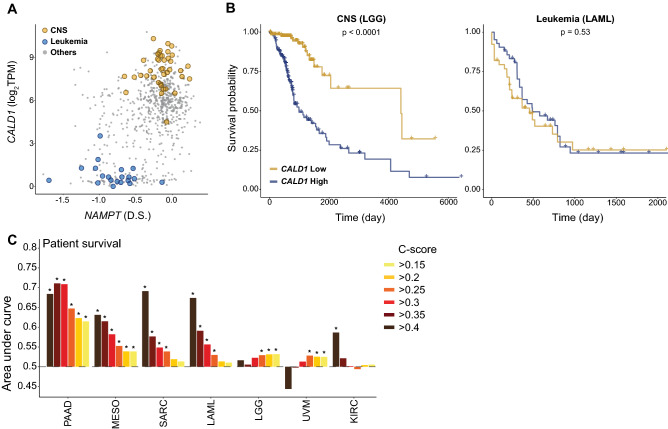
Harnessing cell-specific high C-score gene pairs for cancer patient prognosis. (**A**) C-score plot of *NAMPT* dependency score and *CALD1* gene expression (C-score = 0.447). Yellow circles represent central nervous system (CNS) cell lines and blue circles denote leukemia cell lines. (**B**) Kaplan–Meier overall survival plots for CNS (LGG, lower grade glioma, left panel) and leukemia (LAML, acute myeloid leukemia, right panel) cancer patients. Patients were stratified by high (> 75%) or low (< 25%) expression of *CALD1*, and *p*-values were calculated using Cox regression controlling for age, sex, pathological staging, clinical staging, and tumor grade, and corrected for multiple testing (FDR < 0.1). (**C**) AUC of ROC curves based on C-score gene pair-based prediction of survival for each cancer type with different C-score cutoffs. Only the cancer types with at least one significantly positive C-score cutoff and those containing more than 50 positive genes predicting patient survival with statistical significance are shown (*denotes *p* < 0.05).

Inspired by the proto-oncogenes we identified according to C-scores (Fig. 2E), we wondered if cancers in various tissues may take advantage of the buffering capacities endowed by the CEBU mechanism for robust proliferation. In other words, would higher CEBU-mediated buffering capacity render cancers more robust and aggressive, thereby resulting in a poorer prognosis? To test this hypothesis, we established a “ground-truth” of expression-based cancer patient prognosis by analyzing patient gene expression and survival data for all 30 available cancer types from The Cancer Genome Atlas (TCGA)^45^. Here, we assessed differential patient survival against gene expression using Cox regression and controlling for clinical characteristics including age, sex, pathological stage, clinical stage, and tumor grade, followed by multiple testing correction (FDR < 0.1). Then, we examined the performance of CEBU-mediated buffering capacity in terms of predicting the ground-truth dataset. As an example, in Fig. 6A we present potential buffering to *NAMPT* of the NAD^+^ salvage pathway, where cancers may be addicted to this pathway^46^. We discovered that the *NAMPT-CALD1* gene pair, comprising the *NAMPT* dependency score and *CALD1* gene expression, demonstrate a high C-score of 0.446, and its CEBU-mediated buffering capacity is high in CNS but low in blood cells. When we stratified patients based on *CALD1* expression, we observed a considerable difference in survival for patients suffering lower grade glioma (LGG—a cancer of the CNS, see Supplementary Table S4 for cross-referencing between cell lines and TCGA cancers and for the full names of cancer abbreviations), but not for patients with acute myeloid leukemia (LAML—a cancer of the blood, Fig. 6B left panel for LGG and right panel for LAML). Mean CEBU-mediated buffering capacity for the *NAMPT*:*CALD1* gene pair is 1.47 in the CNS (i.e. tissue/cell types displaying strong buffering capacity), but only − 0.88 in leukemic blood cells (i.e. exhibiting weak buffering capacity) (Fig. 6A). Thus, based on our ground-truth dataset, the buffering capacity of the *NAMPT* and *CALD1* gene pair in different tissue/cell types can be used to predict patient survival for specific cancer types.

We systematically assessed how buffering capacity from C-score-identified gene pairs could help predict cancer patient survival for all 30 TCGA cancer types. We found that for 15 of those cancers, at least 1% of genes across the genome can predict patient survival (with statistical significance assessed by Mann–Whitney U test), and for 7 of these 15 cancer types, the performance of CEBU-mediated buffering capacity for at least one C-score cutoff was significantly better than random (AUC > 0.5, FDR < 0.1) (Fig. 6C). In general, buffering capacity-based predictions performed better for higher C-score cutoffs. Taken together, our results show that the CEBU-mediated buffering capacity derived from our C-score index can be indicative of cancer aggressiveness, as illustrated by patient survival.

## Discussion

In multicellular organisms, different cells and tissues conduct various functions via specialized cellular structures and/or according to specific states (e.g., signaling and/or metabolic states) by regulating cell- and tissue-specific gene expression. Our study proposes that this cell- and tissue-specific gene expression not only contributes directly to tissue-specific functions, but also allows buffering for functional enhancement. This type of functional buffering, which we have termed cell-specific expression buffering (CEBU), suggests essential functions of a gene can be buffered by increased expression of another gene. Accordingly, essentiality of broadly expressed genes, such as housekeeping genes, are coupled with increased expression of another gene, potentially maintaining housekeeping functions in specific tissues. Furthermore, the CEBU relationship appears to be especially prevalent in tissues of low regenerative capacity (e.g., bone and neuronal tissues) and it can stratify cancer patient survival, inferring tumor aggressiveness. Although functional buffering has long been known as critical to biological robustness, the mechanisms underlying functional buffering remain largely unknown^3^. The proposed CEBU concept represents a possible buffering mechanism in multicellular organisms that is critical for tissue homeostasis and cancer robustness.

One key feature of CEBU is the distinct patterns of expression and dependency (essentiality) between the buffered genes (G1s) compared to buffering genes (G2). In general, G1s tend to be broadly expressed with stronger dependency, whereas expression of G2s is more tissue-specific and less essential (Fig. 4A and Supplementary Fig. S2C). Generally, the essentiality of genes is correlated with their expression level and tissue specificity^47–49^. Housekeeping genes that are broadly expressed in most cells exhibit stronger essentiality. In contrast, genes expressed in specific cell types are considered to have weaker essentiality. Here, cooperation of housekeeping genes and tissue-specific genes of similar functions may be associated by the proposed CEBU mechanism, enabling regulation of cellular functions via functional buffering across tissue/cell types. Specifically, house-keeping functions like metabolism, transcription, translation, and cell-cycle-related processes are highly enriched among high C-score gene pairs (Fig. 2E), likely inferring that house-keeping functions can be robustly maintained via CEBU-mediated functional buffering.

As a cell- and tissue-specific buffering mechanism, we postulate that CEBU may endow buffering capacity on specific cells/tissues in order to maintain their functions and survival, such that CEBU may compensate for the lack of regenerative capacity in certain tissues. Our analysis shows neuronal and bone tissues to have the strongest CEBU-mediated buffering capacities (Fig. 4C, 5C), both of which exhibit relatively low regenerative capacities^50–52^. In contrast, human blood cells, which are fully regenerated in 4 to 8 weeks^53^, are predicted to have the weakest buffering capacities (Fig. 5C). Therefore, it is tempting to speculate that cell types of weaker regenerative capacities, such as neurons, need to sustain robust cellular functions through buffering. In contrast, highly regenerative tissues are frequently replaced, so they have less need for functional buffering.

Unlike the needs-based buffering mechanism, whereby the buffering gene is only activated when its buffered function is compromised, the CEBU-mediated intrinsic buffering proposed herein maintains a constitutively active state with cell- and tissue-specificity. Since the buffering gene (G2) is continuously expressed, there is no need for a control system to monitor if a function has been compromised and to activate the expression of the buffering genes. As a result, no response time is needed for intrinsic buffering, unlike for needs-based buffering. Intrinsic buffering thus poses an advantage in its capability to buffer housekeeping genes, which is performed constitutively, differing from the needs-based buffering that is mostly characterized as stress-responsive^54^. Accordingly, the proposed intrinsic CEBU mechanism may enable or adjust buffering capacity by regulating the expression of buffering genes via cell- or tissue-specific epigenetic regulators. Overall then, CEBU describes a simple, efficient and potentially versatile mechanism for functional buffering in humans and potentially other multicellular organisms.

We observed an enrichment of duplicated genes among high C-score gene pairs, supporting the notion that duplicated genes contribute to the context-dependent essentiality of their paralogous genes^10–12^. In addition to duplicated genes, our C-score index identified a high percentage of non-duplicated gene pairs with high buffering capacities (Fig. 2B), and these non-duplicated gene pairs tend to belong to the same pathways and/or protein complexes (Fig. 2C,D). Therefore, it is possible that many of these G1s and G2s represent non-orthologous functional analogs. As support, we identified the *POP7* and *RPP25* gene pair, both are subunits of the ribonuclease P/MRP complex, as potential buffering pairs (Fig. 3C,D, Supplementary Figs. S5D–F, S6). One simple scenario could be that G1 and G2 physically interact with each other to form a protein complex, wherein G1’s function can be structurally substituted by G2. More sophisticated and indirect functional buffering can also occur between G1s and G2s given the complex interactions among biological functions^55^. We expect that CEBU exerts buffering effects through additional types of molecular interactions, which remain to be tested experimentally.

Experimental validations of functional buffering remain to be a challenging task. Buffering of one gene may be achieved through multiple mechanisms from multiple genes, leading to difficulties in selecting test targets and types of experiments. Moreover, functional buffering may be different across cell lines, which is in line with the proposed CEBU mechanism. Using CEBU as a base provides an approach to select for target gene pairs and cell lines and can test for buffering via expression. We have demonstrated the validity of using the C-score plot to identify functional buffering in two pairs of genes, *FAM50A*–*FAM50B* and *POP7*–*RPP25* through shRNA-silencing (Fig. 3, Supplementary Figs. S5, S6). However, the type of experiment remains to be a technical challenge. shRNA-silencing may not fully recapitulate the screening results from CRISPR. We noticed a discrepancy between DepMap screening data (average D.S. of *FAM50B* in A549 is 0.256) and our own validation results regarding the essentiality of *FAM50B* in A549 (Supplementary Fig. S5C). This discrepancy could be due to the incompleteness of shRNA-mediated gene silencing, leaving a low expression level of targeted endogenous genes. This low level of expression may trigger secondary responses that leads to different phenotypic outcomes. Nevertheless, our results showed potential synergistic effects upon dual suppression in predicted sensitive cells lines as oppose to single knockdowns (Fig. 3, Supplementary Figs. S5, S6), though other experimental methods, such as CRISPR, may provide additional validation.

C-score-derived cell-specific buffering capacities comply well with experimentally validated genetic interactions in human cells (Fig. 5B and Supplementary Fig. S8), indicating that CEBU may potentially represent a critical mechanism for synthetic lethality in human cells. However, accurate inference of synthetic lethality is difficult as all computational predictions inevitably generate false positives. Despite a stringent selection with low *p*-values and controls for multiple testing (Fig. 1C), it remains possible that some high C-score gene pairs are resultant of random chance. Validation of synthetic lethality interaction remains a daunting challenge in practice. To systematically characterize genetic interactions in organisms with complex genomes due to large numbers of possible gene pairs, i.e. ~ 200 million gene pairs in humans. Another method to infer authenticity of synthetic lethality is of recurring computational predictions across studies. However, predictions emanating from different studies exhibit little overlap^30^, evidencing the marked complexity of synthetic lethality in humans. It is possible that each of the current predictions focuses on one or a few characteristics of synthetic lethality such that assembling them does not reveal the same predictions. In this sense, the CEBU mechanism proposed here can contribute both experimentally and computationally to a better characterization of human genetic interactions.

Using G2 expression of a high C-score gene pair to stratify cancer patients, we observed a significant difference in cancer patient survival, indicating that stronger CEBU-mediated buffering capacity could be indicative of cancer aggressiveness in patients (see Fig. 6A,B for an example). Indeed, buffering capacity helped predict cancer patient survival in 7 of 15 cancer types and, generally, the performance was better for higher C-score cutoffs (Fig. 6C). This result supports our hypothesis that stronger buffering capacity via higher G2 expression contributes to cancer robustness in terms of proliferation and drug resistance. Prognosis in cancer patients can be affected by multiple factors, and some of them are unmeasurable and not accounted for in current study. However, given the complexity of cancers, it is surprising to see the results possibly suggests a general predictivity of cancer prognosis by individual high C-score gene pairs. We suspect that some cancer cells may adopt this cell- and tissue-specific buffering mechanism to enhance their robustness in proliferation and stress responses by targeting the expression of buffering genes. Clinically, the expression of such buffering genes could represent a unique feature for evaluating cancer progression when applied alongside other currently used clinical characteristics. Finally, experimental validation of C-score-predicted genetic interactions will help identify potential drug targets for tailored combination therapy against specific cancers.

## Methods

### Retrieval and processing of dependency score and gene expression data

Data on dependency scores and CCLE (Cancer Cell Line Encyclopedia) gene expression were downloaded from the DepMap database (DepMap Public 19Q4)^14,15^. Dependency scores modeled from the CERES computational pipeline based on a genome-wide CRISPR loss-of-function screening were selected. CCLE expression data was quantified as log_2_ TPM (Transcripts Per Million) using RSEM (RNA-seq by Expectation Maximization) with a pseudo-count of 1 in the GTEx pipeline (https://gtexportal.org/home/documentationPage). Only uniquely mapped reads in the RNA-seq data were used in the GTEx pipeline. Integrating and cross-referencing of the dependency score and gene expression datasets yielded 18,239 genes and 684 cell lines. Genes lacking dependency scores for any one of the 684 cell lines were discarded from our analyses.

### C-score calculation

Our C-score index integrates the dependency scores of buffered genes (G1) and the gene expression of buffering genes (G2) to determine the buffering relationship between gene pairs. Genes with mean dependency scores > 0 or mean gene expression < 0.5 log_2_ TPM were discarded, yielding 9196 G1s and 13,577 G2s. The C-score integrates the correlation (*ρ*) and slope between the dependency score of gene *G*1 and the gene expression of gene *G*2, defined as:

$$\mathrm{C}{\text{-}}\mathrm{score}={\rho }_{G1,G2}\left(1+b\frac{{slope}_{min}}{{slope}_{G1,G2}}\right),$$

where *ρ* denotes the Pearson correlation coefficient and *slope*_*min*_ denotes the minimum slope of all considered gene pairs that present a statistically significant positive correlation, which is 0.00748 in this analysis. The normalized slope can be weighted by cell- and tissue-type specific *b*. In this analysis, *b* is set as 1 for a pan-cell or pan-cancer analysis.

### Construction of C-score null distribution

As a control, expression of each gene was randomly shuffled amongst the 684 cell lines. The shuffled expression values were used in place of true expression in the C-score calculation to create a randomly shuffled null distribution. Five shuffled expression datasets were generated to calculate five null distributions, one of which is shown.

### Duplicated gene assignment

Information on gene identity was obtained from ENSEMBL (release 98, reference genome GRCh38.p13)^56^. Two genes are considered duplicated genes if they have diverged from the same duplication event.

### Enrichment analysis for buffering gene pairs

For enrichment analysis of duplicated gene pairs, we conducted hypergeometric test. Given a specified C-score cutoff, the test assesses statistical significance by calculating the proportion of duplicated gene pairs higher than cutoff among all duplicated gene pairs, and then compare to the proportions of all high C-score pairs higher than cutoff among all high C-score pairs.

For enrichment analysis of same function or signaling pathways, we adopted a previously described methodology^57^. Briefly, GO and KEGG gene sets were downloaded from the Molecular Signatures Database (https://www.gsea-msigdb.org/gsea/msigdb/). The number of total possible gene pairs is 9196 (G1) × 13,577 (G2). The condition of G1 and G2 being the same gene was excluded as a potential buffering gene pair under all C-score cutoffs. Enrichment was calculated as:

$$\mathrm{log}\frac{\frac{{e}_{ac}}{{e}_{a}} }{\frac{{e}_{c}}{{e}_{t}}},$$

where ${e}_{ac}$ represents the number of gene pairs that are both annotated and with buffering capability, ${e}_{a}$ is the number of annotated gene pairs, ${e}_{c}$ is the number of buffering gene pairs, and ${e}_{t}$ is the total number of gene pairs.

Protein–protein interaction (PPI) data was downloaded from the STRING database (version 11)^39^. Only high-confidence interactions (confidence > 0.7) in human were considered. The STRING database determines confidence by approximating the probability that a link exists between two enzymes in the KEGG database. Data on protein core complexes were downloaded from CORUM (http://mips.helmholtz-muenchen.de/corum). The enrichment calculation is the same as for GO and KEGG, except that ${e}_{ac}$ represents the number of gene pairs that have PPI or are in the same complex and have buffering capability, and ${e}_{a}$ is the number of gene pairs that have PPI or are in the same complex.

### Construction of our human buffering gene network

The directional human buffering gene network was constructed from gene pairs exhibiting high C-scores (> 0.25). For illustration, isolated subnetworks are not shown. We visualized the network using Cytoscape (https://cytoscape.org/) and MATLAB. GO enrichment was conducted on each cluster using g:Profiler^58^. To identify functionally-related gene clusters in the human buffering gene network, the genes with enriched functions were inputted into the SAFE algorithm^59^. The neighbor radius was determined by regional enrichment of sub-networks for each GO-enriched function.

### Experimental validation

A549, HT29, LN18, MCF7, and U2OS cell lines were selected based on their distribution across the C-score plots (Fig. 3A,C), indicating different buffering capacities. All cell lines were purchased from ATCC and they were cultured in Dulbecco’s Modified Eagle Media (LN18), Ham's F-12K Medium (A549), or RPMI 1640 media (HT29, MCF7, and U2OS) supplemented with 5% fetal bovine, serum, 100 U/mL penicillin, 100 μg/mL streptomycin, and 250 ng/mL fungizone (Gemini Bio-Products). Cell growth was monitored by time-lapse imaging using Incucyte Zoom, taking images every 2 h for 2–4 days. To suppress *FAM50A, FAM50B*, *POP7* and *RPP25* expression, lentivirus-based shRNAs were delivered individually or in combination. The gene-specific shRNA sequences are: *FAM50A*-CCAACATTGACAAGAAGTTCT and GAGCTGGTACGAGAAGAACAA; *FAM50B*-CACCTTCTACGACTTCATCAT; *POP7*-CTTCAGGGTCACACCCAAGTA and CGGAGACCCAATGACATTTAT; and *RPP25*-CCAGCGTCCAAGAGGAGCCTA. To ensure better knockdown of gene expression, shRNAs were delivered twice (7 days and 4 days before seeding). Equal numbers of cells were seeded for cell growth measurements by time-lapse imaging using Incucyte Zoom. The lentivirus-based shRNAs were purchased from the RNAi core of Academia Sinica. The growth rate under each condition was measured by fitting cell confluence to an exponential growth curve using the Curve Fitting Toolbox in MATLAB.

### Bliss independence model

Cytotoxic synergy was measured using the Bliss independent model^41^. The Bliss model is presented as a ratio of the expected additive effect to the observed combinatorial effect:

$${E}_{bliss}=\frac{{E}_{A}+{E}_{B}-{E}_{A}\times {E}_{B}}{{E}_{AB}},$$

where *E* is the effect of drug *A*, *B*, or a combination of *A* and *B*. A ratio of lower than one indicates potential synergistic effect, while larger than one indicates no synergistic effect. Effect was measured by the relative cell growth, based on the fold-change of confluency between 0 and final hours upon suppression of *FAM50A* and *FAM50B* or suppression of *POP7* and *RPP25* in all cell lines.

### Cell-specific buffering capacity and comparison to experimental genetic interactions

Cell-specific buffering capacity was derived from the C-score of a given gene pair and gene expression of the buffering gene (G2) in the cell line of interest following the equation:

$$\mathrm{buffering \; capacity}=\frac{\mathrm{ cell \; line \; expression}- 25\mathrm{th \; percentile \; of \; all \; expression }(\mathrm{G}2)}{{slope}_{mod}},$$

$$\mathrm{where \; }{slope}_{mod}=\mathrm{C}{\text{-}}\mathrm{score}\times \frac{\left.sd(G2 \; expression\right)}{\left.sd(G1 \; dependency\right)}$$

where *sd* = standard deviation. The 25th percentile cutoff for expression is determined empirically, although different percentile cutoffs do not qualitatively affect the measurements of buffering capacities (Fig. 5A).

Combinatorial CRISPR screen-derived genetic interaction scores were pooled from four literature sources^19,32,43,44^ (Supplementary Table S3). We only considered cell lines that appear in DepMap CERES 19Q4. There were two C-scores for each gene-pair of the experimental dataset (either gene could be a G1), and we assigned the higher C-score for that gene-pair. Overall, we curated 10,222 genetic interaction scores in various cell lines from the literature, and 1986 out of 10,222 genetic interaction scores had a C-score > 0.1. To evaluate the validity of buffering capacity, we generated a ground-truth dataset by assigning gene-pairs with a positive genetic interaction as false for buffering and a negative genetic interaction as true for buffering. The qualitative performance of buffering capacity against this ground-truth dataset was assessed by ROC curve. Additionally, we correlated the buffering capacity directly via a ground-truth genetic interaction score for quantitative evaluation. We calculated the false discovery rate (FDR) using the Benjamini–Hochberg procedure with a threshold < 0.1.

### Tissue specificity

To calculate tissue-specificity, cell lines were grouped by their respective tissues, and expression of genes in cell lines of the same tissue were averaged. Tissue specificity was calculated as tau (*τ*)^42^, where *τ* is defined as:

$$\tau =\frac{{\sum }_{i-1}^{N}(1-\frac{{x}_{i}}{{x}_{max}})}{N-1},$$

with *N* denoting the number of tissues, *x*_*i*_ denoting the expression of a gene, and *x*_*max*_ denoting the highest gene expression across all tissues. Note, expression values were log-transformed, so log_2_ TPM < 1 was considered as 0 in tissue specificity calculations^60^.

### Cancer-specific survival prediction according to C-score gene pairs

Gene expression and survival data from The Cancer Genome Atlas (TCGA)^45^ was retrieved from Xena^61^. The DepMap cancer cell lines were mapped to TCGA cancers based on the annotation in Supplementary Table S4 (cancers that do not have a matched cancer type in CERES 19Q4 were not analyzed). To systematically analyze cancer prognosis, we first performed a multiple test correction on the *p*-values from Cox regression controlling for age, sex, pathological stage, clinical stage and tumor grade. We calculated the FDR using the Benjamini–Hochberg procedure with a threshold < 0.1. The ground-truth table for each cancer was constructed using the adjusted *p*-value. AUC of ROC curves were used to assess the performance of survival based on buffering capacity. AUCs and ROCs were generated using python and R. The statistical significance of AUC was assessed by Mann–Whitney U test^62^ to evaluate if gene expression with a positive Cox coefficient (poorer prognosis) reflected significantly higher buffering capacities in each cancer with different C-score cut-offs. We excluded the results where there are fewer than 50 positive genes when calculating ROCs. The *p*-values of the Mann–Whitney U test were adjusted using the Benjamini–Hochberg procedure with a threshold < 0.1.

## Supplementary Information

Supplementary Information.

Supplementary Tables.

## Data Availability

All C-score gene pairs (https://figshare.com/s/6f8929c6543687a6062f) and programming code (https://figshare.com/s/b778489bb2f6fc3b0069) are available in the FigShare repository.
