# Supplementary material for: Functional buffering via cell-specific gene expression promotes tissue homeostasis and cancer robustness
Source: Sci Rep. 2022 Feb 22;12:2974. doi: 10.1038/s41598-022-06813-4 (PMC8863889; doi:10.1038/s41598-022-06813-4)
Supplement: Supplementary file 1 — Supplementary Information. [file 41598_2022_6813_MOESM1_ESM.pdf]

Supplementary Methods

Modeling C-score null distribution as a normal distribution

The null distribution fit a normal distribution based on qqplot analysis. The mean and standard deviation of the null distributions were used to model a normal distribution and statistical significance was assessed using a *t*-test.

Control for mean and standard deviation for dependency and expression analysis

To analyze the increase in standard deviation of dependency/expression with increasing C-scores, we controlled for the mean value of dependency/expression. For each gene, 100 other genes with mean dependency/expression differing the least from that gene were selected from the genome. Then, one gene was randomly selected from out of those 100 genes to be the control for mean. The distribution of the standard deviations of the controls was plotted alongside the original distribution. This process was repeated five times to ensure that there was no selection bias during random selection. Only one of the five replicates is shown.

Mutational analysis

Mutation information was retrieved from DepMap. We considered all types of mutations, including hotspot, damaging, and non-conserving mutations. Genes with significant oncopotential[1] to be oncogenes or tumor suppressors were considered cancer-related genes, otherwise they were considered non-cancer-related.

Copy number analysis

Copy number was retrieved from DepMap. We used bootstrapping to estimate the random correlation of copy number variation and expression while controlling for expression mean and standard deviation. Genes were divided into 100 bins based on their expression mean and standard deviation, respectively. Each G2 was randomly replaced by a gene in the same bin. We performed 10,000 randomizations under each C-score cutoff for every G2.

Chromosomal adjacency analysis

Genomic positions of genes were retrieved from ENSEMBL (release 98, reference genome GRCh38.p13). For each G1-G2 pair, the genomic distances of G2 to any of G1 duplicated gene was calculated if they are located on the same chromosome. Any G2-G1 duplicated gene not located on the same chromosome were discarded from the analysis. The genomic distances were calculated from all combinations of gene start and gene end of G2 and that of G1 duplicated genes. Finally, the minimum absolute distances were taken for each G1-G2 pair. As a control, the minimum absolute distances for G1 duplicated gene to a randomly selected gene was calculated using the same method.

RNA isolation, reverse transcription, and quantitative PCR

RNA was isolated using the MACHEREY NAGEL nucleospin RNA kit according to manufacture instructions (MN, REF 740955.50). cDNA was prepared from 2 µg RNA using iScript™ cDNA Synthesis Kit (BioRad, #1708890) in 40 µl reactions. After cDNA generation, the final volume was brought to 160 µl. qPCR was performed in a 20 µl reaction containing 10 µl iQ™ SYBR® Green Supermix (BioRad, #1708880), 4 µl cDNA, 5 µl H2O, and 1 µl of 10 mM specific primers. qPCR was carried out using BioRad CFX96 Touch Real-Time PCR Detection System. The

forward and reverse primers used for qPCR are: *FAM50A* - CGACTTCATCGTCACCAAGGCA and ACTCATCCTTCTCCACAGTGGC; *FAM50B* – AACACGGTGCAGCAGTTCCTGA and GTCGTAGAAGGTGTGGTAGTGC; *POP7* – GCTCTGAGATCTACATTCACGGC and TCCACGGTGGAGGTATTGGCAG; *RPP25* – TTACAGACCGCATACACACAC and CAGTGATTCACAGCCCAAGA; and GAPDH – ACATCGCTCAGACACCATG and TGTAGTTGAGGTCAATGAAGGG.

Western blot analysis

Cell pellets were lysed in RIPA lysis buffer and 30 μg of total lysate were run per lane and separated using 4%-12% gradient gels (ThermoFisher, NP0336BOX). Proteins were transferred to Immobilon-FL PVDF membrane (Millipore, IPFL85R). After blocking and incubation with primary/secondary antibodies, membranes were imaged with Amersham Typhoon. Mouse monoclonal anti-actin was from Millipore (MAB1501, 1:10000). Rabbit polyclonal anti-RPP25 was obtained from ABclonal (A9973, 1:1000). Mouse anti-GAPDH was obtained from Abcam (ab8245, 1:5000). Secondary antibodies anti-Mouse 680 RD and anti-Rabbit 800 CW (Li-Cor) were used at a dilution of 1:30000.

Supplementary Figure and Figure Legends

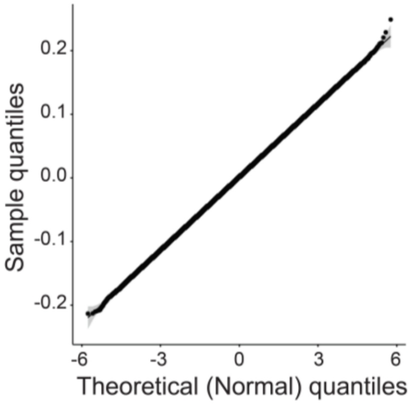

Supplementary Figure 1. Distribution of C-scores

The randomly shuffled distribution represents a normal distribution generated using qqplot with a theoretical normal distribution. One of five qqplot distributions is shown. Range of mean and standard deviation for the five randomly shuffled distributions are 0.001-0.000 and 0.0383-0.0389, respectively.

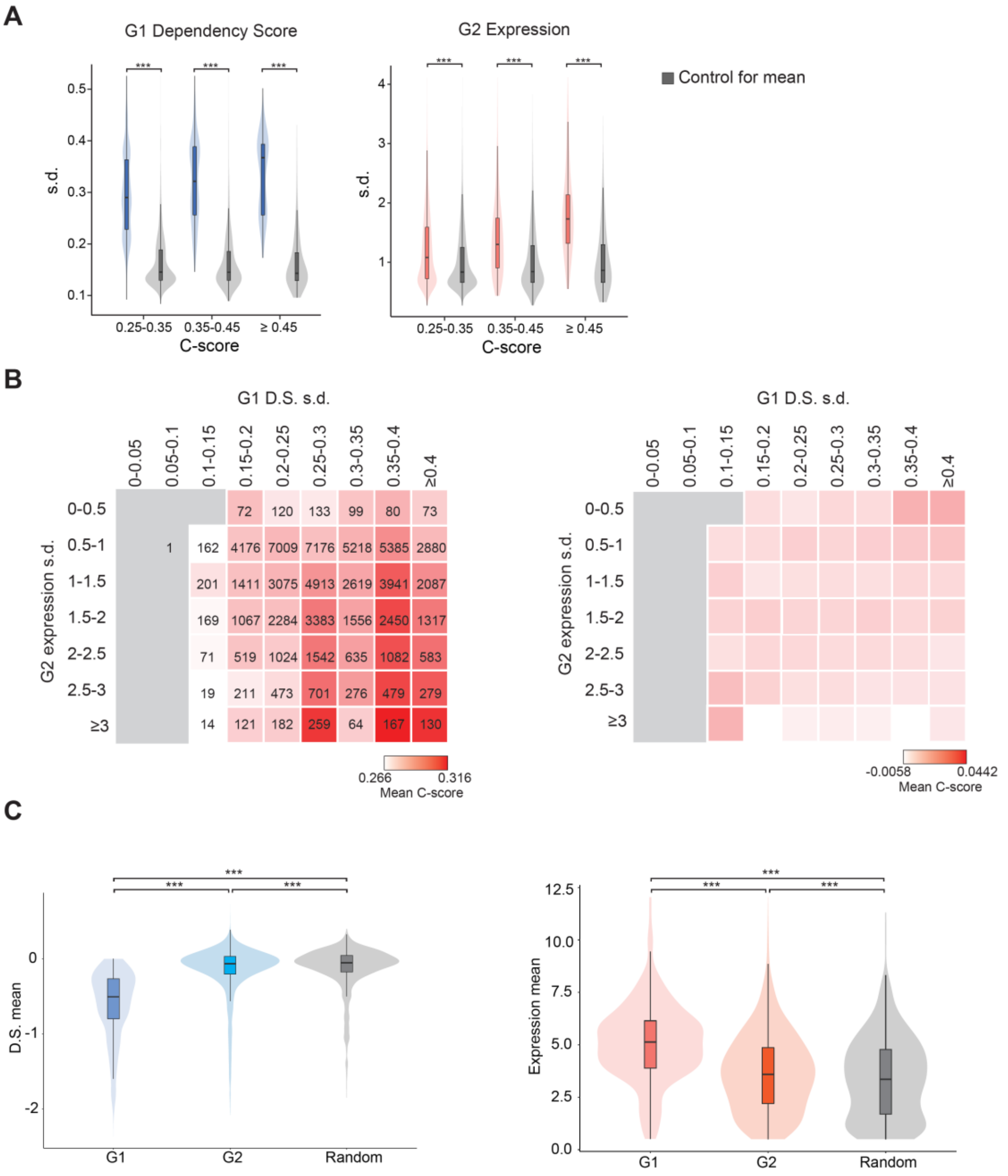

Supplementary Figure 2. Correlation properties of G1 dependency score and G2 gene expression according to increasing C-score

(A) The standard deviation (s.d.) of G1 dependency score (D.S.) and G2 gene expression increases with increasing C-score. Grey represents control distributions for mean values, which were constructed based on the s.d. of randomly selected genes with similar mean values of dependency

or expression (see **Supplementary Methods**). Differences between each C-score interval to the control distribution for mean values are significant based on a one-tailed two-sample *t*-test (*** denotes p-value < 0.001). Five random selections were performed, and the results are comparable. One of the five random selections is shown. (B) Left panel: mean C-score of intervals of increasing s.d. for G1 dependency and for G2 expression values. Numbers of genes in each interval are shown in the cells. Only cells containing more than 10 genes are colored. Right panel: mean C-score of intervals of increasing s.d. of G1 dependency and randomly shuffled G2 expression values. Random shuffling of G2 expression ensured the same mean and s.d. values were retained, but it uncoupled the cell line associations, which acted as a control for the increased s.d. of both G1 dependency and G2 expression with increasing C-score. (C) Mean of dependency score (D.S., blue) and gene expression (red) of G1 and G2. The random plot (grey) was constructed by randomly selecting the same number of genes from the genome (*** statistically significant at $p < 0.001$, based on a one-tailed paired *t*-test for G1 and G2 comparison and one-tailed two-sample *t*-test for comparison with random).

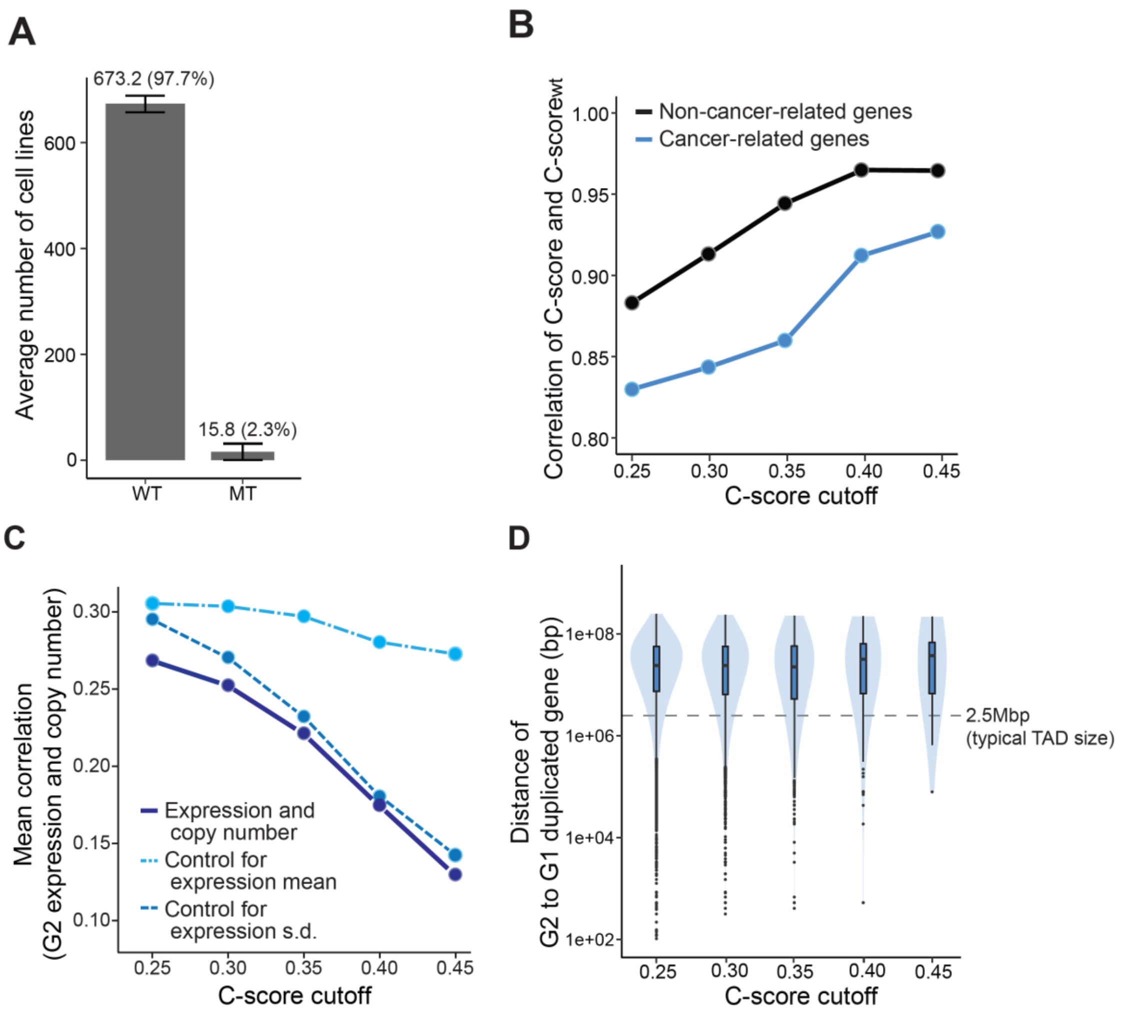

Supplementary Figure 3. Decreasing effects of mutational variation as C-score increases

(A) Average number of wild type or mutated cell lines for each C-score gene pair. (B) Correlation between C-scores and C-scores calculated using only wild-type genes (C-score$_{wt}$) for different C-score cutoffs. Genes were assigned as cancer-related if they have a significant oncopotential[1] to be oncogenes or tumor suppressor genes. Black line represents non-cancer-related genes. Blue line represents cancer-related genes. (C) The mean correlation between G2 copy number and its expression for different C-score cut-offs is represented by the dark blue line. The lighter blue dashed lines are correlation controls for G2 expression mean and standard deviation. Controlling for G2 expression mean and standard deviation revealed that standard deviation is the major

contributor to the diminished correlation between G2 expression and CNV. (D) The absolute minimum distance of G2 to G1 duplicated gene as C-score increases. The grey dashed line shows 2.5 Mbp, which is a typical size of topological associated domains (TAD).

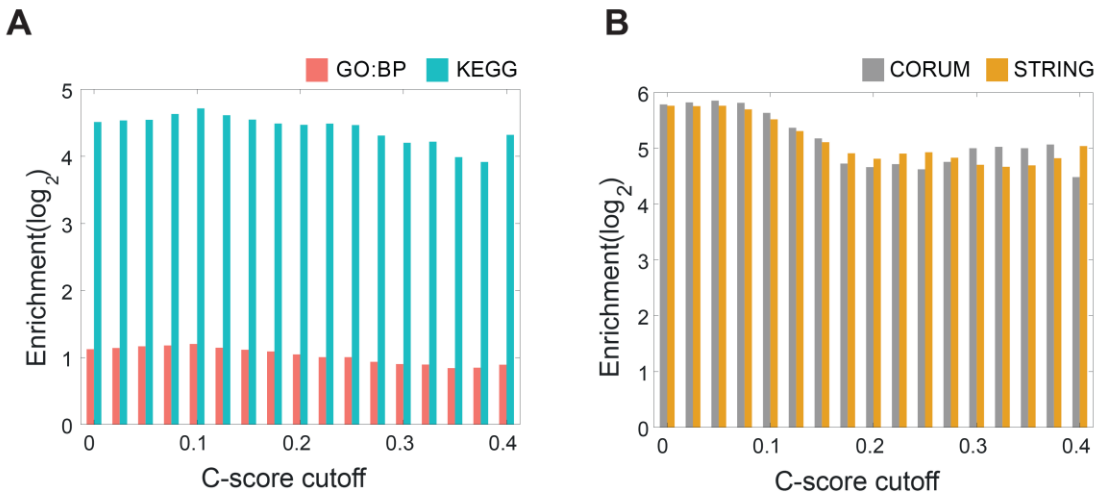

Supplementary Figure 4. Functional and pathway enrichments of C-score-identified duplicated genes

(A-B) Functional enrichment of C-score gene pairs with increasing C-score cutoff for duplicated genes. (A) Pairs of duplicated genes annotated with the same gene ontology biological process (GO:BP) term or KEGG pathway are enriched in C-score gene pairs, and enrichment remains relatively constant with increasing C-score cut-off. (B) Pairs of duplicated genes with annotated protein-protein interactions from STRING and within the same protein complex from CORUM are enriched in C-score gene pairs, and enrichment remains relatively constant with increasing C-score cut-off.

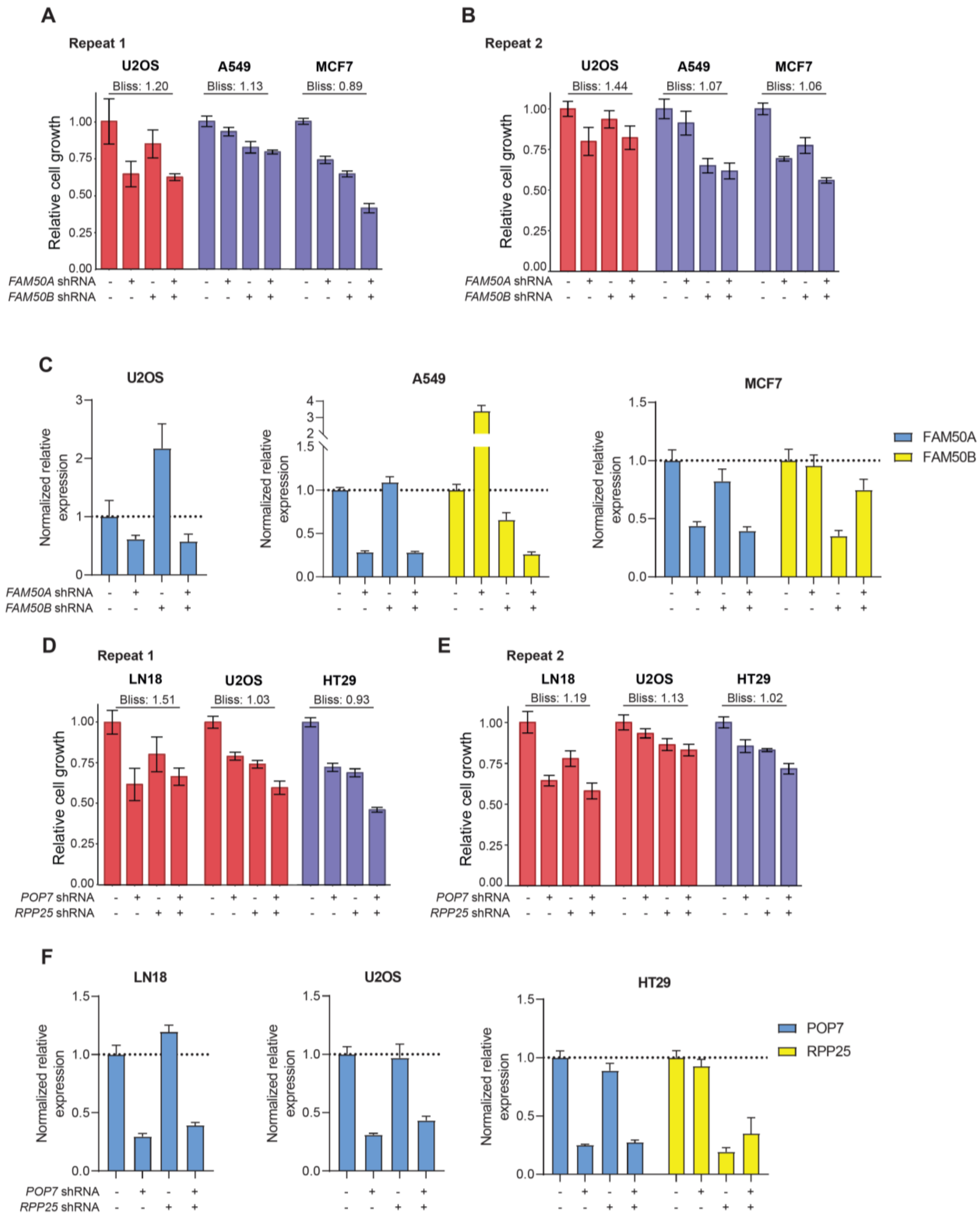
A
Repeat 1
U2OS
Bliss: 1.20
A549
Bliss: 1.13
MCF7
Bliss: 0.89
Relative cell growth
FAM50A shRNA
FAM50B shRNA
B
Repeat 2
U2OS
Bliss: 1.44
A549
Bliss: 1.07
MCF7
Bliss: 1.06
C
U2OS
A549
MCF7
Normalized relative expression
FAM50A
FAM50B
D
Repeat 1
LN18
Bliss: 1.51
U2OS
Bliss: 1.03
HT29
Bliss: 0.93
POP7 shRNA
RPP25 shRNA
E
Repeat 2
LN18
Bliss: 1.19
U2OS
Bliss: 1.13
HT29
Bliss: 1.02
F
LN18
U2OS
HT29
POP7
RPP25

Supplementary Figure 5. Experimental validation of cell-specific expression buffering between *FAM50A* and *FAM50B*, and *POP7* and *RPP25*

(A-B) Relative cell growth based on fold-change in confluency of the U2OS, A549, and MCF7 cell lines with or without *FAM50A* or *FAM50B* suppression. Bliss scores indicate strength of synergy between double suppression of *FAM50A* and *FAM50B* compared to either gene alone. Error bars indicate standard deviation of six technical repeats. (A) Repeat 1. Cell growth in U2OS, A549, and MCF7 were collected from 0 to 90 hours. (B) Repeat 2. Cell growth in U2OS and A549 were collected from 0 to 72 hours. Cell growth in MCF7 was collected from 0 to 96 hours. (C) Gene expression as measured by real-time RT-qPCR and normalized to *GAPDH* mRNA expression for repeat 1 of *FAM50A* and *FAM50B*. Fold change in expression relative to shCtrl (control shRNA infected) was shown and plotted as mean ±SEM (n=4). (D-E) Relative cell growth based on fold-change in confluency of the HT29, U2OS and LN18 cell lines with or without shRNA-based *POP7* or *RPP25* suppression. Bliss scores indicate strength of synergy between double suppression of *POP7* and *RPP25* compared to either gene alone. Error bars indicate standard deviation of six technical repeats. (D) Repeat 1. Cell growth were collected from 0 to 90 hours. (E) Repeat 2. Cell growth were collected from 0 to 72 hours. (F) Gene expression as measured by real-time RT-qPCR and normalized to *GAPDH* mRNA expression for repeat 1 of *POP7* and *RPP25*. Fold change in expression relative to shCtrl (control shRNA infected) was shown and plotted as mean ±SEM (n=4). (F) Western blot analysis of *RPP25* expression in LN18, U2OS, and HT29 cells 4 days after shRNA infection. *GAPDH*, Actin, and Ponceau S, serve as internal controls.

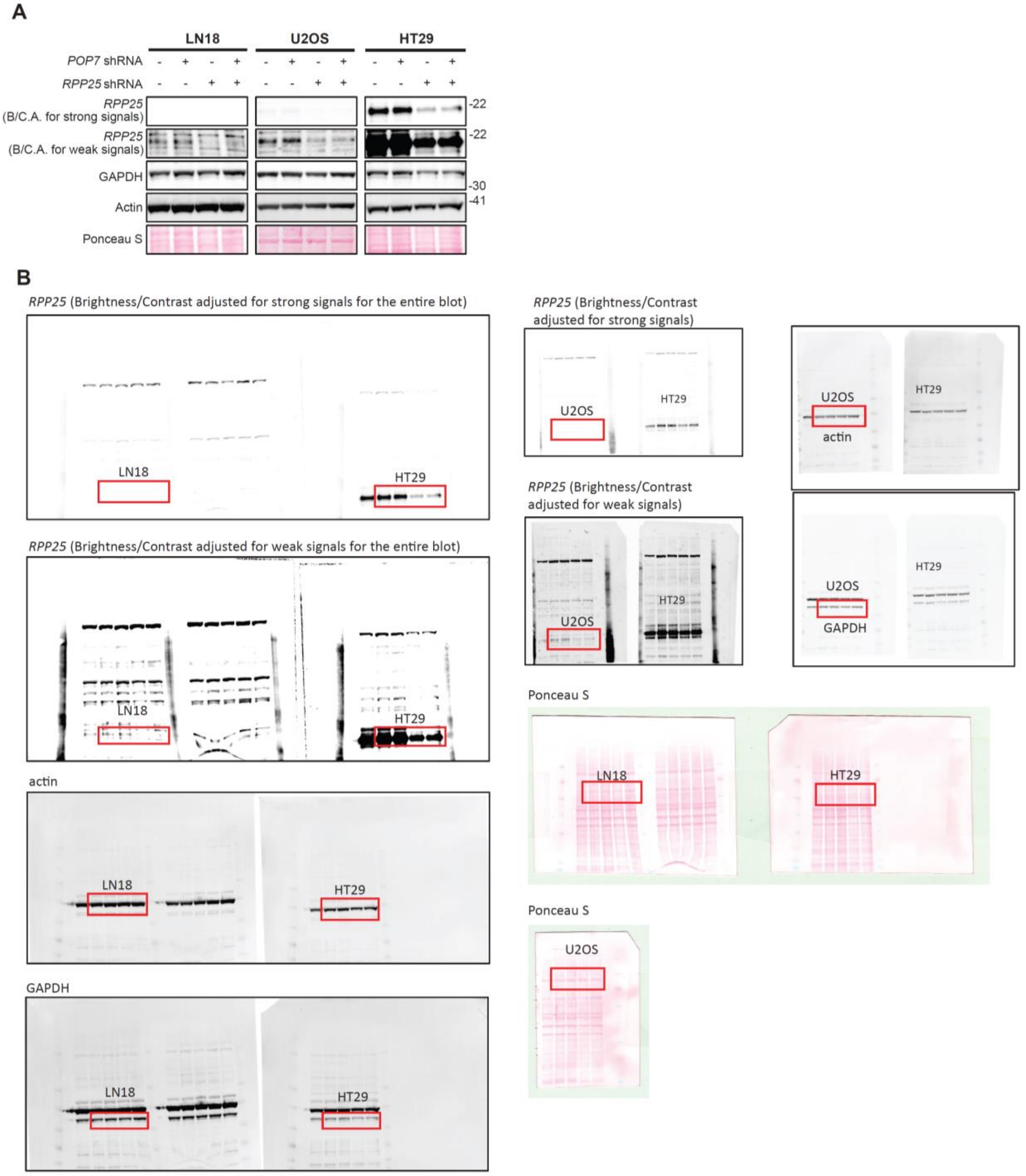
A
LN18
U2OS
HT29
POP7 shRNA
RPP25 shRNA
RPP25 (B/C.A. for strong signals)
RPP25 (B/C.A. for weak signals)
GAPDH
Actin
Ponceau S
-22
-22
-30
-41
B
RPP25 (Brightness/Contrast adjusted for strong signals for the entire blot)
RPP25 (Brightness/Contrast adjusted for weak signals for the entire blot)
actin
GAPDH
RPP25 (Brightness/Contrast adjusted for strong signals)
RPP25 (Brightness/Contrast adjusted for weak signals)
Ponceau S
Ponceau S

C

RPP25 - LN18 and HT29

Original blot
Intensity:
Min displayed value: 19
Max displayed value: 65534

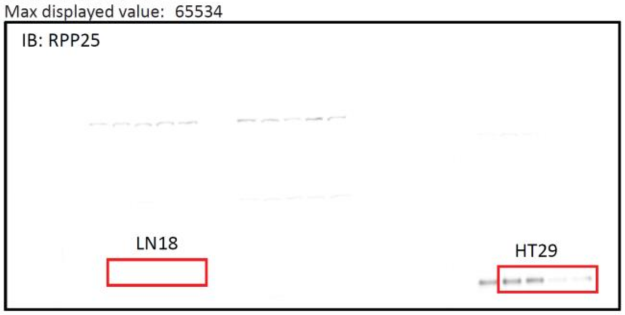

Brightness/Contrast adjusted by ImageJ
Intensity:
Min displayed value: 274
Max displayed value: 13326

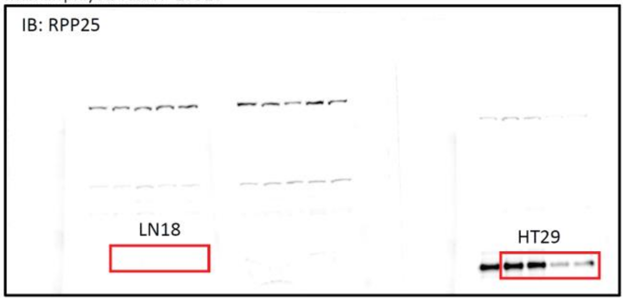

Brightness/Contrast adjusted by ImageJ
Intensity:
Min displayed value: 274
Max displayed value: 2834

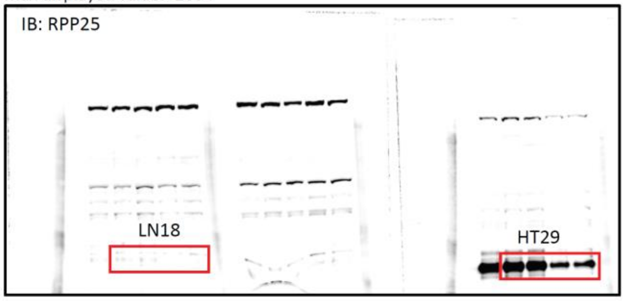

Brightness/Contrast adjusted by ImageJ
Intensity:
Min displayed value: 274
Max displayed value: 784

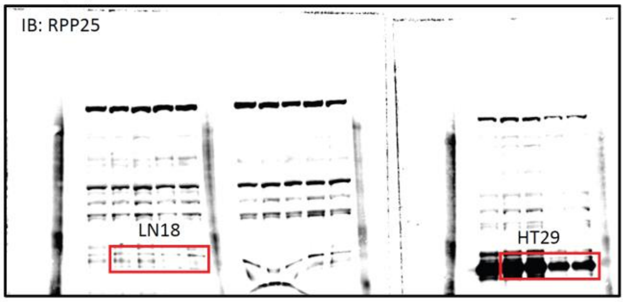

RPP25 - U2OS and HT29

Original blot
Intensity:
Min displayed value: 16
Max displayed value: 65534

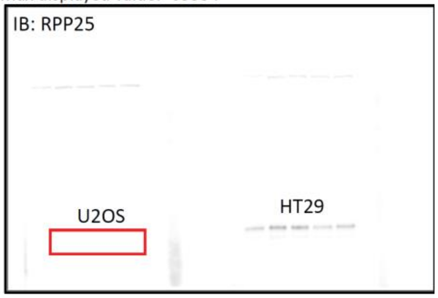

Brightness/Contrast adjusted by ImageJ
Intensity:
Min displayed value: 527
Max displayed value: 13068

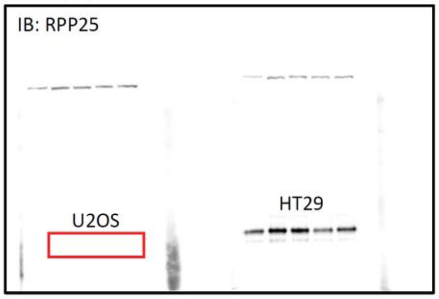

Brightness/Contrast adjusted by ImageJ
Intensity:
Min displayed value: 0
Max displayed value: 4149

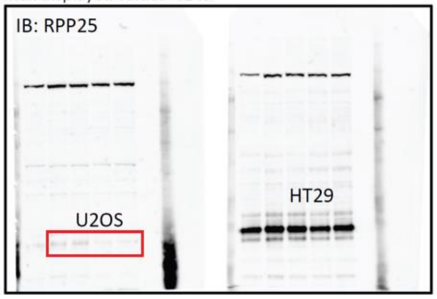

Brightness/Contrast adjusted by ImageJ
Intensity:
Min displayed value: 0
Max displayed value: 1418

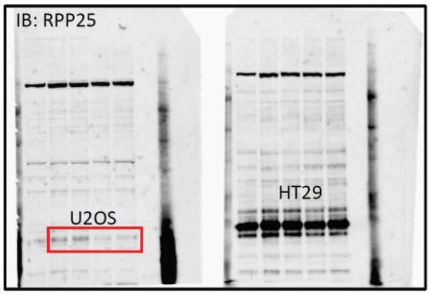

Supplementary Figure 6. Western blot analysis of cell-specific expression buffering between *FAM50A* and *FAM50B*, and *POP7* and *RPP25*

(A) Western blot analysis of *RPP25* expression in LN18, U2OS, and HT29 cells 4 days after shRNA infection. *GAPDH*, Actin, and Ponceau S, serve as internal controls. B/C.A., Brightness/contrast adjusted. (B) Full length blots. Image processing (brightness/contrast adjustment) was applied equally across the entire image. The expression level of *RPP25* in HT29 was used as a reference for the brightness and contrast adjustment to allow better visualization of the weak *RPP25* expression in LN18 and U2OS cells. (C) Progression of level of intensity in brightness/contrast adjustments leading up to the brightness/contrast adjusted results in (A) and (B). The levels of endogenous *RPP25* in LN18 and U2OS are low, so brightness and contrast adjustments are unavoidable. The original blots and three different levels of intensities in brightness/contrast adjustments by ImageJ (https://imagej.nih.gov/ij/) are shown. The bottom adjustments were presented in (A) and (B). Notably, though the RPP25 blots for LN18 and HT29 were run on separate gels, they were imaged at the same time under the same acquisition conditions. The same approach is done for the *RPP25* blots for U2OS and HT29.

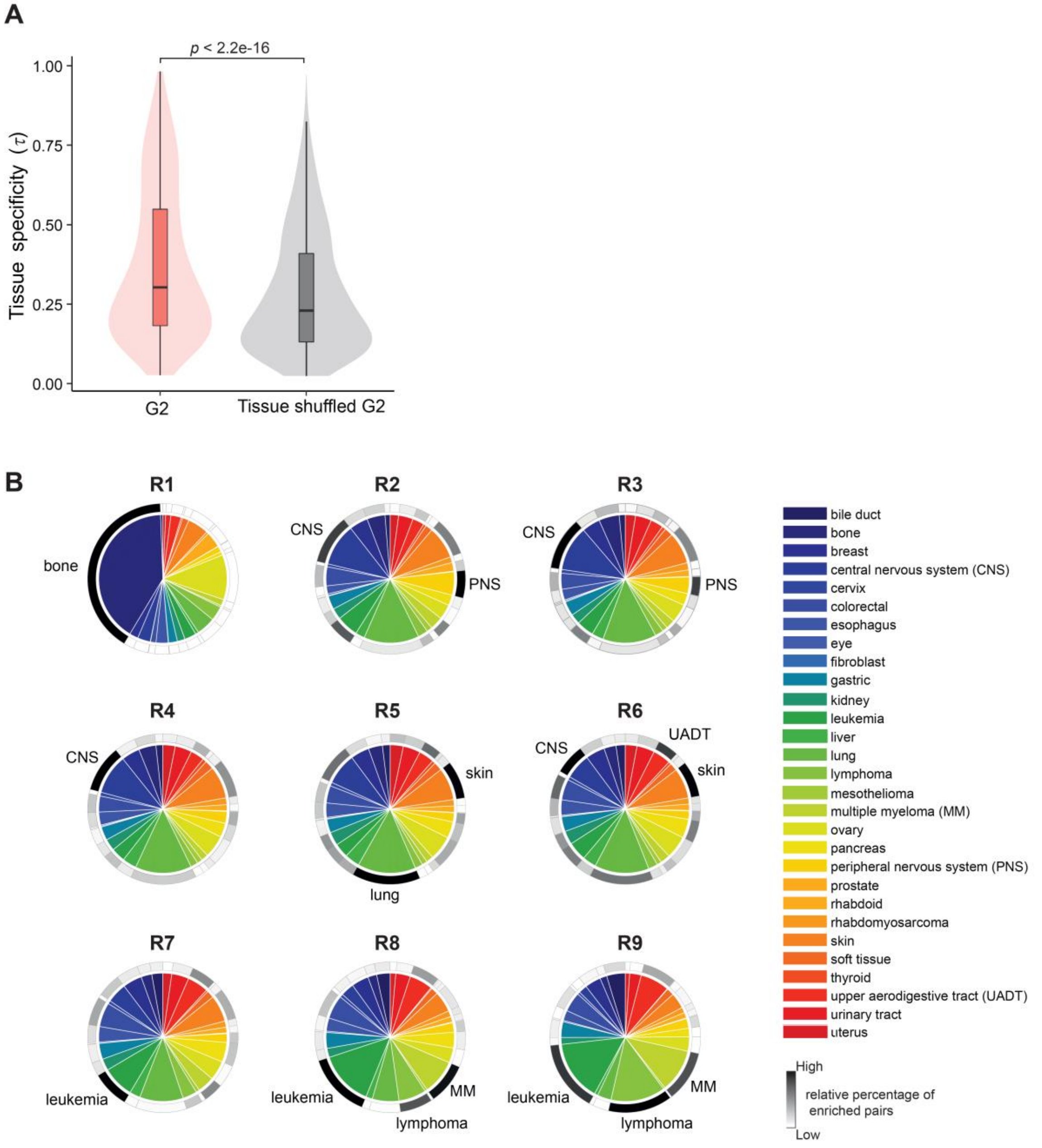

Supplementary Figure 7. Control for tissue specificity of CEBU and proportions of tissue type for each region

(A) Tissue specificity (τ) of G2s and after we had randomly shuffled the tissues for the cell lines associated with G2s. τ was calculated for tissue-shuffled G2s from high C-score gene pairs. Statistical significance was assessed by paired-t test. (B) Tissue/cell-type specificity of each region

(R1-R9) of the normalized C-score plot. The colored pie charts indicate the proportion of each tissue/cell type in each of the regions. The greyscale rings around the pie charts represent the relative percentage of statistically enriched gene pairs for the corresponding tissue/cell types. The tissue/cell types with high percentages of enriched gene pairs (dark grey or black) are annotated for each region.

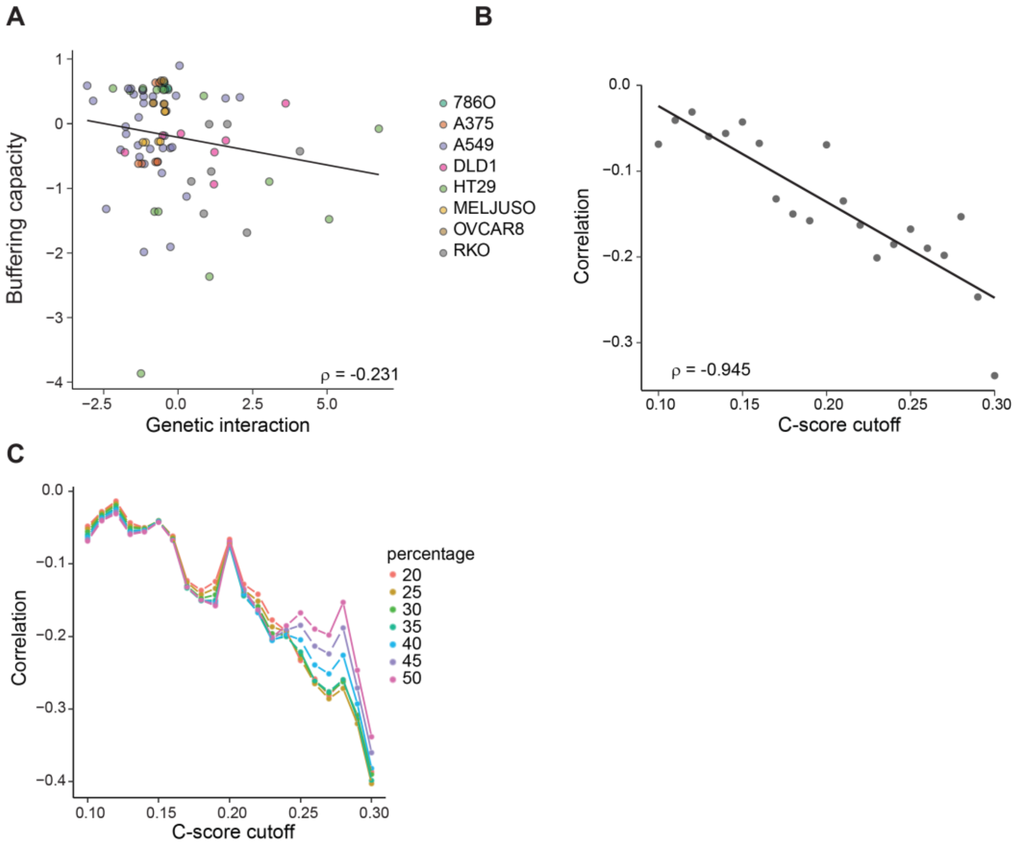

Supplementary Figure 8. C-score-based prediction of cell-specific genetic interaction using buffering capacity

(A) Correlation ($\rho = -0.231$, p = 0.034, n = 84) between existing published genetic interactions (see **Table S3** for detailed information) and our predicted buffering capacities with a C-score cutoff of 0.25. (B) Concordance ($\rho = -0.945$, p < 0.05) between different C-score cutoffs and the correlation (shown in A) between existing published genetic interactions and our predicted buffering capacities. (C) Effect of varying the G2 expression percentile cutoff (from 20% to 50%) on the correlations between our buffering capacities and experimental results according to different C-score cut-offs.

Supplementary References

1 Davoli, T. *et al.* Cumulative haploinsufficiency and triplosensitivity drive aneuploidy patterns and shape the cancer genome. *Cell* **155**, 948-962, doi:10.1016/j.cell.2013.10.011 (2013).
